# The Effectiveness and Safety of First-Line Thioguanine in Thiopurine-Naïve Inflammatory Bowel Disease Patients

**DOI:** 10.1093/ibd/izad197

**Published:** 2023-09-02

**Authors:** Femke Crouwel, Ahmed B Bayoumy, Chris J J Mulder, Job H C Peters, Paul J Boekema, Luc J J Derijks, Sybrand Y de Boer, Paul C van de Meeberg, Ishfaq Ahmad, Hans J C Buiter, Nanne K de Boer

**Affiliations:** Department of Gastroenterology and Hepatology, Amsterdam Gastroenterology Endocrinology Metabolism Research Institute, Amsterdam University Medical Centre, Vrije Universiteit Amsterdam, Amsterdam, the Netherlands; Department of Gastroenterology and Hepatology, Amsterdam Gastroenterology Endocrinology Metabolism Research Institute, Amsterdam University Medical Centre, Vrije Universiteit Amsterdam, Amsterdam, the Netherlands; Department of Gastroenterology and Hepatology, Amsterdam Gastroenterology Endocrinology Metabolism Research Institute, Amsterdam University Medical Centre, Vrije Universiteit Amsterdam, Amsterdam, the Netherlands; Department of Gastroenterology and Hepatology, Rode Kruis hospital, Beverwijk, the Netherlands; Department of Gastroenterology and Hepatology, Máxima Medical Centre, Veldhoven, the Netherlands; Department of Clinical Pharmacy and Pharmacology, Máxima Medical Centre, Veldhoven, the Netherlands; Department of Gastroenterology and Hepatology, Slingeland Hospital, Doetinchem, the Netherlands; Department of Gastroenterology and Hepatology, Slingeland Hospital, Doetinchem, the Netherlands; Department of Gastroenterology and Hepatology, Streekziekenhuis Koningin Beatrix, Winterswijk, the Netherlands; Department of Radiology and Nuclear Medicine, Amsterdam University Medical Centre, Vrije Universiteit Amsterdam, Amsterdam, the Netherlands; Department of Gastroenterology and Hepatology, Amsterdam Gastroenterology Endocrinology Metabolism Research Institute, Amsterdam University Medical Centre, Vrije Universiteit Amsterdam, Amsterdam, the Netherlands

**Keywords:** thioguanine, inflammatory bowel disease, clinical effectiveness

## Abstract

**Background:**

Currently thioguanine is solely used as treatment for inflammatory bowel disease after azathioprine and/or mercaptopurine failure. This study aimed to determine the safety, effectiveness, and 12-month drug survival of thioguanine in thiopurine-naïve patients with inflammatory bowel disease.

**Methods:**

A retrospective cohort study was performed in thiopurine-naïve patients with inflammatory bowel disease treated with thioguanine as first thiopurine derivate. Clinical effectiveness was defined as the continuation of thioguanine without the (re)initiation of concurrent biological therapy, systemic corticosteroids, or a surgical intervention. All adverse events were categorized by the Common Terminology Criteria for Adverse Events.

**Results:**

A total of 114 patients (male 39%, Crohn’s disease 53%) were included with a median treatment duration of 25 months and a median thioguanine dosage of 20 mg/d. Clinical effectiveness at 12 months was observed in 53% of patients, and 78% of these responding patients remained responsive until the end of follow-up. During the entire follow-up period, 26 patients were primary nonresponders, 8 had a secondary loss of response, and 11 patients were unable to cease therapy with systemic corticosteroids within 6 months and were therefore classified as nonresponders. After 12 months, thioguanine was still used by 86% of patients. Fifty (44%) patients developed adverse events (grade 1 or 2) and 9 (8%) patients ceased therapy due to the occurrence of adverse events. An infection was documented in 3 patients, none of them requiring hospitalization and pancytopenia occurred in 2 other patients. No signs of nodular regenerative hyperplasia or portal hypertension were observed.

**Conclusions:**

At 12 months, first-line thioguanine therapy was clinically effective in 53% of thiopurine-naïve inflammatory bowel disease patients with an acceptable safety profile.

**Key Messages**

**What is already known?**

Thioguanine is solely used as maintenance treatment for inflammatory bowel disease after azathioprine and/or mercaptopurine failure.

**What is new here?**

Thioguanine therapy was clinically effective in 53% of thiopurine-naïve inflammatory bowel disease patients at 12 months with an acceptable safety profile and low (8%) cessation rate related to adverse events.

**How can this study help patient care?**

Given the effectiveness and tolerability, thioguanine can potentially play a role as first-line maintenance therapy for inflammatory bowel diseases instead of conventional thiopurines.

## Introduction

Azathioprine and mercaptopurine are well-established (maintenance) drugs in the management of both Crohn’s disease (CD) and ulcerative colitis (UC).^1-3^ Although they are effective in the maintenance of remission, up to 40% of patients have to discontinue therapy, mainly due to the development of adverse events.^4^ Especially in patients who develop adverse events, switching to the alternative thiopurine derivate thioguanine is an option.^5^ In contrast to both azathioprine and mercaptopurine, thioguanine has a less complicated metabolism and is directly converted by HGPRT (hypoxanthine-guanine phosphoribosyltransferase) into the pharmacologically active 6-thioguaninenucleotides (6-TGNs) without the formation of potential toxic metabolites like 6-MMP (6-methylmercaptopurine).^6^ Thioguanine has shown promising therapeutic results in the treatment of inflammatory bowel disease(IBD) and seems to be effective and tolerated in up to 65% of patients who failed previous therapy with azathioprine/mercaptopurine.^5,7-10^ Therefore, thioguanine was recently registered in the Netherlands as a certified IBD treatment for patients who failed azathioprine/mercaptopurine therapy. Due to the efficacy and safety of thioguanine after azathioprine/mercaptopurine failure, one could speculate that thioguanine would also be an effective and safe primary treatment option for thiopurine-naïve IBD patients, although data are lacking. The aim of this study was to evaluate the safety, efficacy, and 12-month drug tolerability of off-label thioguanine in azathioprine/mercaptopurine-naïve IBD patients.

## Methods

### Ethical Considerations

Ethical approval was obtained by the Medical Ethics Review Committee of VU University Medical Centre (file number: 2020.457), and the study was conducted in accordance with the principles of the Declaration of Helsinki. Written informed consent was obtained from all participants.

### Study Design and Patient Population

A retrospective multicenter cohort study was performed in 5 centers in the Netherlands (Máxima Medical Centre, Slingeland Hospital, Streekziekenhuis Koningin Beatrix, Rode Kruis Hospital, and Medical Centre de Veluwe) and patients were recruited from January 2011 to March 2022. Data were retrospectively collected from prospectively maintained local databases. Depending on the center, patients were identified by using local hospital pharmacy dispensing records, by using the hospital electronic health software, or by the treating physician. Patients diagnosed with UC, CD or IBD unclassified and treated with thioguanine as first thiopurine derivate aimed at treating IBD were included. Patients who started thioguanine as concomitant immunomodulation during biological therapy were excluded. The decision to prescribe thioguanine as first thiopurine-derivate was made by the treating physician.

### Data Collection

Patient and disease characteristics, drug history, and clinical, biochemical, radiological, and histopathological data were retrieved from the patients’ medical record. Disease was classified according to the Montreal classification.^11^ When available, 6-TGN concentrations in red blood cells, determined using the method as described by Dervieux and Boulieu,^12^ were recorded. Thiopurine S-methyltransfersase (TPMT) genotyping was performed at the Máxima Medical Centre and based on the method by Schütz et al.^13^ Endoscopies performed prior to thioguanine initiation and during therapy and all abdominal ultrasonography, magnetic resonance imaging, computed tomography (CT), and liver biopsies performed during or after thioguanine initiation were assessed. When available, the physician global assessment (PGA) at month 12 was recorded.

### Outcomes and Definitions

The primary outcome of this study was clinical effectiveness at 12 months and drug tolerability.

Secondary outcomes included medication-related adverse events, infections, and clinical signs of noncirrhotic portal hypertension such as thrombocytopenia, splenomegaly, or varices.

Clinical effectiveness was defined as the continuation of thioguanine without the (re)initiation of concurrent biological therapy, systemic corticosteroids (ie, prednisone, prednisolone), or a IBD-related surgical intervention. Primary nonresponse was assigned when the criteria for clinical efficacy were not met within 6 months after initiation of thioguanine. Secondary nonresponse was defined as a loss of response after 6 months of thioguanine therapy after an initial response. For corticosteroid-induced remission, clinical effectiveness was only met if oral corticosteroids (including oral budesonide and oral beclomethasone) were withdrawn within 6 months. Initiation of concurrent biologicals, (re)initiating systemic corticosteroids (ie, prednisone, prednisolone), or IBD-related surgical intervention during thioguanine treatment was considered a nonresponse to therapy.

Adverse events were defined as signs or symptoms that occurred after initiation of thioguanine, whether or not the event was considered thioguanine related. Both infections and treatment-related adverse events were classified according to the Common Terminology Criteria for Adverse Events (CTCAE) (version 5.0). According to the CTCAE criteria, grade 1 elevated liver enzymes that spontaneously resolved were also classified as adverse events. Clinically relevant hepatoxicity was defined as a grade 2 toxicity of at least 1 of the following liver enzymes: aspartate aminotransferase, alanine aminotransferase, alkaline phosphatase, γ-glutamyl transferase, or total bilirubin according to the CTCAE, or a grade 1 toxicity if it led to a change in treatment.

Drug survival was determined by the number of patients who continued thioguanine after the initiation, including patients who (re)started biologicals or corticosteroids or underwent an IBD-related surgery.

### Statistical Considerations

Statistical analyses were performed with the use of IBM SPSS 28.0. Categorical variables were presented as number and percentage. Continuous variables were presented as mean ± SD or as median and interquartile range (IQR), depending on their distribution. Normality was tested by visual inspection of histograms and with the Shapiro-Wilk test. The association of gender, IBD type, disease localization, disease behavior, and smoking status on effectiveness rates at 12 months was evaluated with a univariate logistic regression analysis. Patients who did not reach the 12-month follow-up period were considered censored cases from this evaluation. A Kaplan-Meier plot was performed to assess the thioguanine drug survival. A *P* value <0.05 was considered statistically significant.

## Results

### Patient Characteristics

In total, 126 IBD patients treated with thioguanine as first thiopurine derivate were identified. Twelve of them were treated with concomitant biologicals and therefore excluded for further analysis. Of the 114 patients treated with thioguanine therapy without concomitant biologicals, 44 patients were male (39%), 60 (53%) had CD, 51 (45%) had UC, and 3 (3%) had IBD unclassified. Median age at IBD diagnosis and initiation of thioguanine was 34 (IQR, 22-51) and 42 (IQR, 26-57) years, respectively. Ninety percent of patients used comedication at the start of thioguanine (n = 103). Thioguanine was started for various reasons in these patients: induction of remission (12%), maintenance of remission (28%), initiated as maintenance therapy concurrent with (oral or topical/rectal) induction treatment (55%), or steroid dependency (4%). Additional patient and disease characteristics are shown in Table 1.

**Table 1.** Patient characteristics (N = 114).

Age at starting TG, y	42 (26-57)
Sex	
Female	70 (61)
Male	44 (39)
Disease duration, y	3 (1-8)
Inflammatory bowel disease subtype	
Ulcerative colitis	51 (45)
Crohn’s disease	60 (53)
IBD undetermined	3 (3)
Montreal Classification (UC)	
E1: proctitis	3 (6)
E2: left-sided colitis	26 (51)
E3: extensive colitis	22 (43)
Montreal Classification (CD)	
Age at diagnosis	
A1 (≤16 y)	1 (2)
A2 (17-40 y)	35 (58)
A3 (≥40 y)	23 (38)
Missing	1 (2)
Disease location	
L1: only terminal ileum	21 (35)
L2: only colon	20 (33)
L3: ileum and colon	18 (30)
+L4: locations proximal of ileum	1 (2)
+P: perianal disease	6 (10)
Missing	1 (2)
Behavior	
B1: nonstricturing, nonpenetrating	49 (82)
B2: stricturing	8 (13)
B3: penetrating	2 (3)
Missing	1 (2)
Previous bowel surgery	6 (5)
Smoking status	
Current	16 (14)
Former	22 (19)
Never	62 (54)
Missing	14 (12)
Comedication at the start of TG	
Corticosteroids	43 (38)
Corticosteroid enema	10 (9)
Mesalazine enema	4 (4)
Mesalazine suppository	2 (2)
Mesalazine	48 (42)
Budesonide	33 (29)
Combination enema (mesalazine + corticosteroids)	9 (8)
None	11 (10)

Values are median (interquartile range) or n (%).

Abbreviations: CD, Crohn’s disease; TG, thioguanine; UC, ulcerative colitis.

### Clinical Effectiveness

Clinical effectiveness at 6 and 12 months of thioguanine therapy was observed in 64% (n = 72 of 112) and 53% (n = 55 of 104) of patients, respectively. Two patients did not yet reach the 6-month follow-up period and 10 patients did not yet reach the 12-month follow-up period with thioguanine monotherapy but were in corticosteroid-free remission at time of data collection.

The reasons for clinical ineffectiveness of monotherapy TG at month 12 were primary nonresponse (n = 26 of 104 [25%]), a secondary loss of response (n = 3 of 104 [3%]), or the inability to cease therapy with systemic corticosteroids within 6 months (n = 11 of 104 [11%]). Four patients ceased monotherapy thioguanine due the occurrence of adverse events (n = 4 of 104 [4%]), 4 due to patient preference (n = 4 of 104 [4%]), and 1 due to remission (n = 1 of 104 [1%]). Twenty-eight patients with clinical ineffectiveness at month 12 started with anti-tumor necrosis factor (anti-TNF) and 27 of them continued their treatment with thioguanine after the start of anti-TNF.

Of the responding patients at 12 months, 78% (n = 43 of 55) remained responsive until the end of follow-up (median follow-up period 31 [IQR 22-47] months), 5 patients had a secondary loss of response, and 7 of the responding patients at 12 months discontinued thioguanine monotherapy due to side effects (n = 5), patient preference (n = 1), or remission (n = 1).

All 11 patients that were unable to cease therapy with corticosteroids within 6 months continued monotherapy thioguanine, and 2 of them achieved corticosteroid-free clinical remission with monotherapy thioguanine at month 12.

Most patients with clinical effectiveness at month 12 were also in clinical remission according to the PGA scores, and only 2 patients had mild disease according to the PGA. Clinical effectiveness at month 12 was observed in 46% of female and 63% of male patients (odds ratio [OR], 0.49, 95% confidence interval [CI], 0.22-1.102) and in 61% of patients diagnosed with UC and 45% of patients diagnosed with CD (OR, 1.87, 95% CI, 0.84-4.13). In patients diagnosed with UC, there seemed to be no difference in 12-month clinical effectiveness between patients with solely left-sided colitis (67%) and patients with pancolitis (58%) (OR, 1.46 95% CI, 0.42-5.05). Also, in patients with CD, the disease localization and the presence of penetrating/stenosing disease did not seem to be associated with the 12-month clinical effectiveness of thioguanine monotherapy (Supplementary Appendix 1).

### Drug Survival Analysis

**Figure 1.**
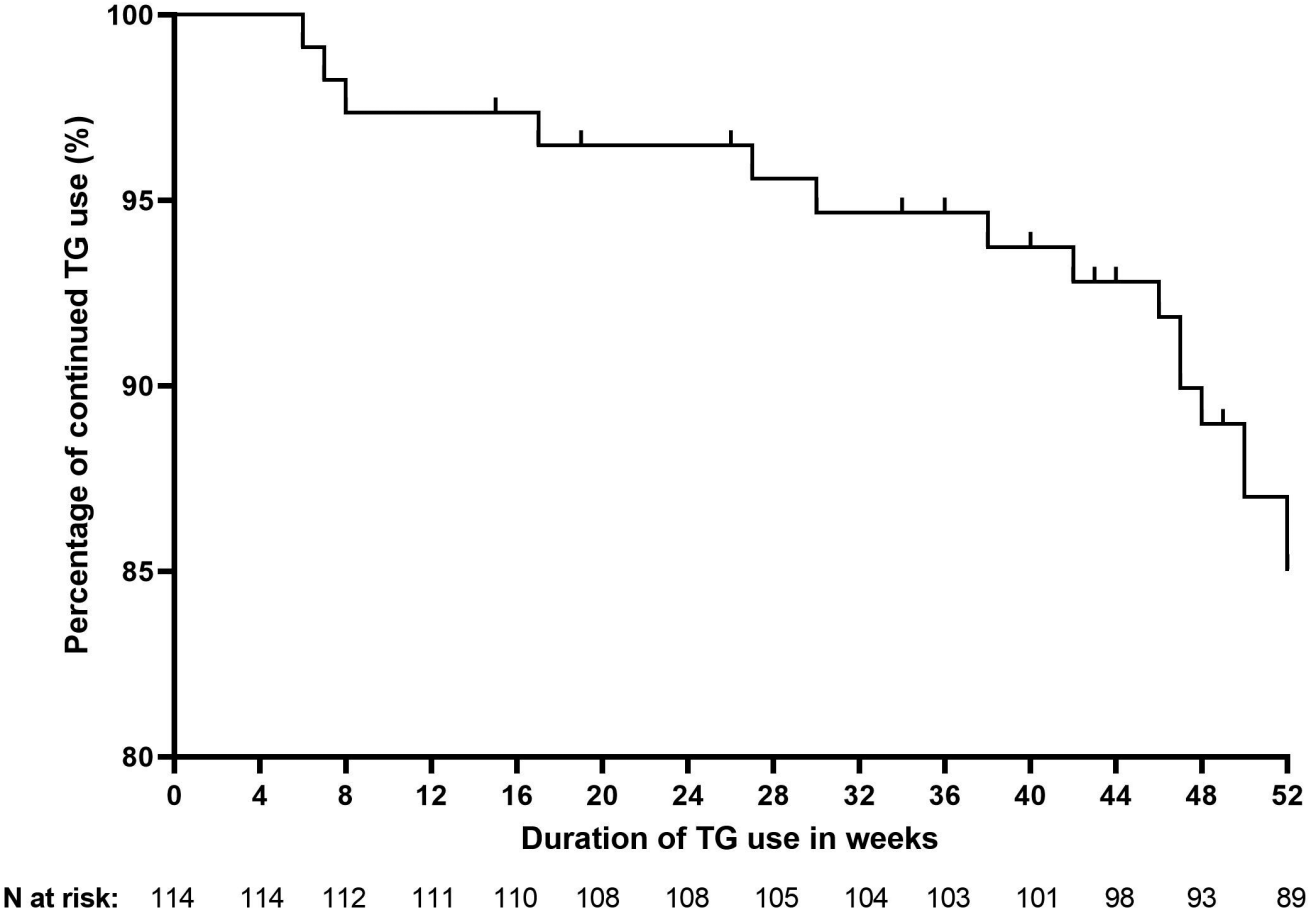
Kaplan-Meier survival curve showing the time to thioguanine (TG) withdrawal for the first 52 weeks.

The median duration of thioguanine (monotherapy or combined with anti-TNF) use of the entire cohort was 25 (IQR, 12-36) months. The proportion of patients who continued thioguanine for the first 52 weeks is depicted in a Kaplan-Meier drug survival curve in Figure 1.

Twelve months after initiation, thioguanine (monotherapy or combined with anti-TNF) was still used by 89 (86%) of 103 patients. Therapy with thioguanine was discontinued in 14 patients before the 12-month follow-up period due to intolerance or adverse events (n = 4), at the patient’s own request (n = 4), due to insufficient therapeutic response (n = 1), due to long-term remission (n = 1), or due to immunomodulator withdrawal from anti-TNF therapy (n = 4). Eleven patients did not yet reach the 12-month follow-up period with either thioguanine monotherapy or combined with anti-TNF therapy at the time of data collection.

During the entire follow-up period (median 27 [IQR, 14-41] months), 69% used thioguanine until the end of follow-up, either as monotherapy or combined with anti-TNF. Thirty-five (31%) patients discontinued therapy with thioguanine due to intolerance or adverse events (n = 9), at patient’s own request (n = 6), due to long-term remission (n = 2), due to insufficient therapeutic response (n = 3), or due to immunomodulator withdrawal from anti-TNF therapy (n = 15). Sixty-two (54%) patients continued monotherapy thioguanine until the end of follow-up, irrespective of clinical effectiveness.

### Adverse Events

In 50 (44%) patients, a total of 99 adverse events were reported (Table 2). Several patients reported more than 1 adverse event. All adverse events were graded according to CTCAE and were mild (73%) or moderate (27%) (Table 2). None of the patients developed grade 3, 4, or 5 adverse events (ie, severe but not immediately life-threatening, life-threatening, or death) during thioguanine treatment. Most patients reported gastrointestinal complaints (n = 16 of 114 [14%]) and/or developed elevated liver enzymes (n = 26 of 114 [23%]), the latter was considered clinically relevant in 9 (8%) patients. One patient developed pancreatitis during treatment with thioguanine, and both alcohol and gallstones were ruled out as potential causative factors. The treating physician stated in his notes that the observed pancreatitis was possibly caused by thioguanine, although other causes such as autoimmune pancreatitis were not ruled out. Therapy with thioguanine was continued in this patient without the reoccurrence of a pancreatitis. None of the patients needed to be admitted for an adverse event.

**Table 2.** Adverse events (n = 99), listed and graded according to the Common Terminology Criteria for Adverse Events

Adverse event	Grade 1 (mild) (n = 72)		Grade 2 (moderate) (n = 27)	
	Subtype	n	Subtype	n
**Blood and lymphatic system disorder**	Anemia	2	Other, pancytopenia	2
**Eye disorders**	Dry eye	1	—	—
**Gastrointestinal disorders**	Bloating	1	Gastroesophageal reflux disease	3
	Dyspepsia	2	Mucositis oral	1
	Gastroesophageal reflux disease	1	Pancreatitis	1
	Gastrointestinal pain	1		
	Nausea	5		
	Vomiting	1		
**General disorders and administration site conditions**	Fatigue	3	Fatigue	2
	Fever	1	Flu like symptoms	1
	Flu like symptoms	1	Malaise	1
**Hepatobiliary disorders**	Other, hepatic steatosis	1	—	—
**Infections and infestations**	Vaginal infection	1	Hepatitis viral	1
			Urinary tract infection	1
**Injury, poisoning, and procedural complications**	Bruising	1	—	—
**Investigations**	Elevated liver enzymes	25	CPK increased	1
	Hypophosphatemia	1	Elevated liver enzymes	1
	Platelet count decreased	1	Lipase increased	1
	White blood cell decreased	5	Platelet count decreased	1
			White blood cell decreased	1
**Musculoskeletal and connective tissue disorders**	Back pain	1	Myalgia	3
	Muscle cramp	2		
	Myalgia	2		
**Nervous system disorders**	Dizziness	1	—	—
**Respiratory, thoracic, and mediastinal disorders**	Cough	1	—	—
**Skin and subcutaneous tissue disorders**	Alopecia	2	Dry skin	1
	Hair color changes	1	Other, hidradenitis suppurativa	1
	Other, hidradenitis suppurativa	1	Other, periorificial dermatitis	1
	Other, mycosis	1	Rash not specified	1
	Pruritus	1		
	Purpura	1		
	Rash acneiform	2		
	Rash not specified	1		
	Skin hypopigmentation	1		
**Vascular disorders**	—	—	Thromboembolic event	2

Adverse events leading to cessation of thioguanine therapy were reported in 9 (8%) patients and included thrombocytopenia (n = 1, grade 2), leukopenia (n = 1, grade 2), pancytopenia (n = 1, grade 2), elevated liver enzymes (n = 3, grade 1), myalgia (n = 1, grade 2), nausea (n = 1, grade 1), and malaise (n = 1, grade 2).

Infections occurred in 3 (2.6%) patients and consisted of a vaginal infection in a 25-year-old woman, a urinary tract infection in a 53-year-old patient, and a 35-year-old patient experienced cytomegalovirus hepatitis during thioguanine treatment. Treatment with thioguanine was only permanently discontinued in the cytomegalovirus patient. None of the patients required hospitalization as a result of the occurred infections.

Two patients (63 and 62 years of age) developed pancytopenia during therapy with thioguanine, leading to a dose reduction followed by cessation of therapy in 1 patient. TPMT genotyping was not performed; however 6-TGN levels were 2900 and 140 pmol/8 × 10^8^ red blood cells (RBC), respectively.

There were 2 patients with a thrombocytopenia, a potential sign of (noncirrhotic) portal hypertension, in our cohort, but in both patients the platelet count rapidly normalized after a cessation or dose reduction of thioguanine. An abdominal ultrasound of the liver was performed in 16 (14%) patients at a median follow-up of 18 (IQR, 4-29) months for various indications: elevated liver enzymes (n = 7), thrombocytopenia (n = 1), hepatitis (n = 1), unexplained fever (n = 1), pancreatitis (n = 1), and unknown (n = 5). Six (37.5%) patients had no abnormalities, 8 (50%) had signs of steatosis, 1 (6%) had liver cysts, and 1 (6%) had a hemangioma. One patient underwent magnetic resonance imaging of the liver for further specification of a liver lesion, which turned out to be a hemangioma. In 4 patients, no liver abnormalities were described at a magnetic resonance enterography. None of the included patients had any signs of (noncirrhotic) portal hypertension (eg, splenomegaly, gastroesophageal varices) or underwent a liver biopsy during follow-up. None of the patients were diagnosed with nodular regenerative hyperplasia (NRH) of the liver in our cohort during the entire follow-up period.

There were no malignancies reported in our cohort during the entire follow-up period.

### Therapeutic Drug Monitoring

Median steady-state (between 4 months and 12 months of therapy) 6-TGN concentrations were available in 49 (43%) patients. The median 6-TGN concentration in the entire cohort was 730 (IQR, 421-1261) pmol/8 × 10^8^ RBC, with median 6-TGN levels in the 12-month clinical responders of 776 (IQR, 460-1162) pmol/8 × 10^8^ RBC and 793 (IQR, 411-1292) pmol/8 × 10^8^ RBC in the nonresponders.

*TPMT* genotyping was performed in 8 patients, and most (87.5%) had the TPMT*1/*1 (wild-type) genotype and therefore had normal *TPMT* enzyme activity. Only 1 patient had a heterozygous *TPMT* genotype (*TPMT* *1/*2, intermediate activity); this patient was treated with 20 mg/d thioguanine, leading to a median 6-TGN level of 727 pmol/8 × 10^8^ RBC without the occurrence of adverse events.

## Discussion

In this study, we reported on the efficacy and safety of thioguanine as first thiopurine derivate in thiopurine-naïve IBD patients. Clinical effectiveness at 12 months of primary thioguanine use was observed in 53% of patients, and 86% were still using thioguanine 12 months after initiation. Adverse events were documented in 44% of patients and were graded as mild or moderate according to CTCAE. Adverse events leading to the withdrawal of thioguanine occurred in only 8% of patients.

Recent studies about the effectiveness of azathioprine/mercaptopurine demonstrated that 1 year after thiopurine initiation, 69% of CD patients and 76% of UC patients did not require treatment escalation to surgery or biological therapy.^14^ A comparable result was demonstrated by a Dutch retrospective study including 1016 patients treated with a thiopurine (710 azathioprine, 302 mercaptopurine, 4 thioguanine), in which 64% of patients did not require hospitalization or treatment escalation to biologicals, corticosteroids, or surgery at month 12.^15^ In contrast to our study, in the first study nothing was reported about the use of concomitant corticosteroids at the start of therapy, and initiation of systemic corticosteroids was not considered a treatment failure, which could potentially overestimate the effect.^14^ In the second study, patients were allowed to switch to thioguanine (32%) in the follow-up period; moreover, patients who discontinued treatment within 12 weeks after initiation were not considered treatment failures.^15^ A recently published prospective placebo-controlled study demonstrated that 51.7% of mercaptopurine users achieved corticosteroid-free clinical remission (ie, Mayo rectal bleeding score = 0 and Mayo stool frequency score = 0 or 1) at week 52.^3^ Corticosteroid-free combined clinical remission and endoscopic improvement at week 52 was achieved by 48.3% of patients.^3^ The largest study to date about the effectiveness of thioguanine after the failure of azathioprine/mercaptopurine reported a sustained clinical effectiveness during 12 months in about 70% of the primary responders and 50% of the total cohort.^8^ The first prospective study demonstrated that 45% of patients treated with monotherapy thioguanine after failure of azathioprine/mercaptopurine achieved sustained corticosteroid-free clinical remission (Harvey-Bradshaw index ≤4 or Simple Clinical Colitis Activity Index ≤2) at month 12.^10^ These results about the effectiveness of both azathioprine/mercaptopurine and second-line thioguanine are broadly in line with the 12-month corticosteroid-free clinical effectiveness rate of 53% observed in our cohort.

Even though 44% of patients in our cohort developed an adverse event, the majority of adverse events were mild and led to cessation of therapy in only 8%, which is lower than the intolerance rate (up to 30%) observed during azathioprine/mercaptopurine therapy.^15-19^ Also, a recent prospective study demonstrated that 20.7% of patients discontinued mercaptopurine due to adverse events.^3^ We speculate that the lower cessation rate related to adverse events in our cohort should be explained by the use of thioguanine instead of azathioprine/mercaptopurine, as the first bypasses several metabolic steps leading to a reduction of potentially toxic thiopurine metabolites.^6^ This speculation is supported by several studies that demonstrated that patients intolerant for azathioprine/mercaptopurine were able to tolerate subsequent therapy with thioguanine.^5,7-10^

Besides switching to thioguanine, another option for patients experiencing adverse events with azathioprine/mercaptopurine is the optimization of therapy with concomitant allopurinol. Several studies demonstrated that switching azathioprine monotherapy to low-dose azathioprine (25%-33% of the original dose) with allopurinol (100 mg) cotherapy leads to an increase in the pharmacological active 6-TGN levels and decrease in the potentially toxic 6-MMP levels and improves both efficacy and tolerability.^20-24^ Therefore, one might speculate that azathioprine combined with allopurinol can, comparable to thioguanine, be an effective first-line therapy for thiopurine-naïve IBD patients. One retrospective study demonstrated indeed a significant higher 12-month clinical benefit rate (54% vs 37%) and a lower adverse event cessation rate (26% vs 45%) with first-line azathioprine combined with allopurinol compared with azathioprine monotherapy.^25^ Nevertheless, when indirectly comparing the data of this study with our cohort, the adverse event–related cessation rate seems to be higher in the patients treated with first-line azathioprine combined with allopurinol compared with our cohort of first-line thioguanine.^25^ Also, a recent prospective study demonstrated that even though 43% of patients in the first-line azathioprine combined with allopurinol group were in clinical corticosteroid and infliximab-free remission compared with 21% in the azathioprine monotherapy group, adverse events leading to withdrawal of therapy occurred in a considerable number of patients undergoing treatment with first-line azathioprine combined with allopurinol (30%).^26^ This may suggest that in thiopurine-naïve IBD patients the direct conversion of thioguanine into 6-TGNs may lead to a better tolerability than optimizing the more complex azathioprine/mercaptopurine pathway by adding allopurinol. A randomized controlled trial is necessary in azathioprine/mercaptopurine-naïve patients, especially considering that a comparative analysis of thioguanine vs azathioprine/allopurinol in patients failing azathioprine/mercaptopurine did not report a difference in the clinical response rate or discontinuation rate due to adverse events.^27^

One of the feared complications during thioguanine therapy is the occurrence of NRH and associated portal hypertension. However, in contrast to earlier observations, thioguanine-induced NRH seems to be dose related (or 6-TGN related) and rarely occurs at the dosage of 20 mg/d normally used in IBD treatment.^28-31^ Furthermore, NRH was recognized in 6% of perioperative liver biopsies taken from thiopurine-naïve IBD patients needing surgery and in up to 2.6% of the general population demonstrated by autopsy studies.^32-34^ There were 2 patients with a thrombocytopenia, a potential sign of (noncirrhotic) portal hypertension, in our cohort, but in both patients the platelet count rapidly normalized after a cessation or dose reduction of thioguanine, so it was likely dose related and not caused by an underlying portal hypertension.

Our study reports on the clinical effectiveness and safety of thioguanine in azathioprine/mercaptopurine-naïve IBD patients in a retrospective fashion, and as a result we need to acknowledge several limitations. All data were retrospectively retrieved from medical records by which information bias could have been introduced, and we also recognize the possibility of missed and therefore unreported cases. It was not possible to formally assess mucosal response and clinical symptoms, as too few endoscopic reports and disease activity indices (Harvey-Bradshaw Index or Simple Clinical Colitis Activity Index) were available. Fecal calprotectin and other laboratory results were not performed at fixed time intervals, making it difficult to compare the results between patients, and because 6-TGN levels and TPMT were not available in the majority of patients, the correlation between these levels and clinical effectiveness was performed on a relatively small number of patients. NUDT15 genotyping was not performed in our cohort. Even though our study had a very strict definition of corticosteroid-free remission, no data were collected about a short course of oral budesonide or oral beclomethasone during monotherapy thioguanine, and therefore only patients who started therapy with oral or intravenously prednisone were classified as thioguanine failures. Nevertheless, our definition of clinical ineffectiveness when corticosteroids were started was comparable to the one used in the aforementioned Dutch retrospective study in which ineffectiveness was, among other criteria, defined as no course of systemic corticosteroids (either oral or intravenously; excluding budesonide, beclomethasone, and locally acting steroids).^15^ Also, the first Dutch prospective study about the effectiveness of thioguanine after conventional thiopurine failure allowed a short course of corticosteroids to extend induction, reintroduce remission, and evade surgery or therapy escalation.^10^

## Conclusions

At 12 months, first-line thioguanine therapy yielded clinical effectiveness rates similar to that of standard azathioprine/mercaptopurine therapy. However, adverse events were mainly mild, and the discontinuation rate related to adverse events (8%) was lower than reported in literature during standard azathioprine/mercaptopurine therapy. The potential role of thioguanine as first-line maintenance therapy for IBD should be studied in a larger prospective and controlled fashion.

## Supplementary data

Supplementary data is available at *Inflammatory Bowel Diseases* online.

izad197_suppl_Supplementary_Material

## Data Availability

The data underlying this article will be shared on reasonable request to the corresponding author.
