# Supplementary material for: The Effectiveness and Safety of First-Line Thioguanine in Thiopurine-Naïve Inflammatory Bowel Disease Patients
Source: Inflamm Bowel Dis. 2023 Sep 2;30(9):1492–9. doi: 10.1093/ibd/izad197 (PMC11369064; doi:10.1093/ibd/izad197)
Supplement: izad197_suppl_Supplementary_Material [file izad197_suppl_supplementary_material.docx]

| ***Possible predictors*** | ***OR*** | ***95% CI*** | ***P -value*** |
| --- | --- | --- | --- |
| **Gender**   - Male - Female | Ref  0.492 | 0.220 - 1.102 | 0.085 |
| **Type of IBD**   - CD - UC | Ref  1.867 | 0.843 – 4.134 | 0.124 |
| **Localization of UC**   - E1: proctitis - E2: left-sided - E3: extensive | 0.364  1.455  Ref | 0.028 – 4.739  0.419 – 5.051 | 0.44  0.56 |
| **Localization of CD**   - Ileum - Colon - Ileocolonic | Ref  0.802  1.768 | 0.218 – 2.952  0.461 – 6.775 | 0.74  0.41 |
| **Disease behavior in CD**   - Inflammatory - Penetrating/Stenosing | Ref  1.180 | 0.314 – 4.431 | 0.81 |
| **Smoking**   - Current - Former - Never | 0.519  0.485  Ref | 0.168 – 1.598  0.168 – 1.397 | 0.25  0.18 |

**Supplementary Table 1.** Univariate logistic regression analysis of patient characteristics affecting clinical effectiveness at month 12 of monotherapy thioguanine

OR, Odds ratio; CI, confidence interval; CD, Crohn’s disease; UC, ulcerative colitis; Ref, reference category
